# What is known about patients’ quality of life with Phenylketonuria and their caregivers? A scoping review

**DOI:** 10.1186/s13023-024-03422-4

**Published:** 2024-10-28

**Authors:** Eduardo Remor, Kamilla Mueller Gabe, Katia Irie Teruya, Ida Vanessa Doederlein Schwartz

**Affiliations:** 1 Graduate Program in Psychology, Institute of Psychology, Social Work, Health and Human Communication, Universidade Federal do Rio Grande do Sul, Porto Alegre, Brazil; 2 Graduate Program in Psychology, Universidade Federal do Rio Grande do Sul, Porto Alegre, Brazil; 3 Department of Genetics, Universidade Federal do Rio Grande do Sul, Porto Alegre, Brazil; 4 Medical Genetics Service, Hospital de Clinicas de Porto Alegre, Porto Alegre, Brazil

**Keywords:** Phenylketonuria, Quality of life, Patients, Parents, Scoping review

## Abstract

**Background:**

Phenylketonuria (PKU) is a rare genetic disorder characterized by a deficiency in the metabolism of the essential amino acid phenylalanine, which has a neurotoxic effect at high concentrations. The available treatment for PKU involves limiting the intake of phenylalanine through a restrictive diet. Strict adherence to treatment is essential for a child’s proper development. Owing to their rare and chronic condition, PKU patients and their caregivers need to address many specific challenges, which can affect their quality of life (QoL).

**Purpose:**

This review aimed to identify, characterize, map, and summarize existing knowledge about the quality of life of PKU patients and their primary caregivers.

**Methods:**

A scoping review was conducted following the PRISMA-ScR guidelines. The PubMed, PsycINFO, EMBASE, Scopus, CINAHL, and BVS databases were searched, and articles published between January 2000 and February 2023 were included.

**Results:**

The search resulted in 3249 articles, 29 of which were selected for analysis. Most studies were cross-sectional, and the highest concentration of publications ranged between 2011 and 2021. Generic self-report questionnaires were the tools most commonly used to assess patients’ and their caregivers’ QoL. A significant negative impact on QoL was found in most studies with pediatric patients and caregivers. High current and lifetime blood Phe levels were associated with worse QoL in several domains, and higher tolerance of ingested phenylalanine was associated with a lower impact on QoL. Among caregivers, psychosocial variables such as stress, anxiety, depression, and child behavior problems were associated with poorer QoL. Higher perceived social and emotional support was a protective factor of QoL in caregivers.

**Conclusion:**

Patients of pediatric age and their caregivers, younger caregivers, and female patients and caregivers seem to be especially vulnerable to QoL impairments. The social and emotional dimensions were the most affected. These results emphasize the importance of combining generic and disease-specific assessment tools to achieve a comprehensive assessment. Despite the growing interest in this topic, the longitudinal literature is limited, and there is a lack of interventional studies on this population. Future interventions addressing diet management and providing psychosocial support may benefit the QoL of the PKU population.

**Supplementary Information:**

The online version contains supplementary material available at 10.1186/s13023-024-03422-4.

Phenylketonuria (PKU; OMIM, 2616000) is a rare genetic disorder caused by an inborn error of metabolism (IEM), a set of diseases characterized by deficient activity in some structures involved in the metabolism process. In PKU, phenylalanine hydroxylase, an enzyme responsible for converting phenylalanine (Phe) to tyrosine, is deficient or absent. Phenylalanine is an essential amino acid found in almost all existing proteins, such as meat, milk, fish, eggs, seeds, and flour. Because of the difficulty in metabolizing Phe, individuals with phenylketonuria have high concentrations of phenylalanine in their blood, which can cause severe and irreversible brain damage due to the neurotoxic effects of excess Phe, such as intellectual disability, microcephaly, motor disorders, and seizures [1, 2]. Globally, the prevalence of phenylketonuria is 1:10,000 newborns, which varies by country and ethnic group. In Brazil, an incidence of 1:15,000 to 1:25,000 is estimated [3].

The diagnosis is made through neonatal screening during the first days of the baby’s life. Once detected, immediate treatment adherence is needed to protect the PKU child from neurological damage, preferably before ten days of age. PKU can be categorized as classic or mild on the basis of the concentration of Phe in the blood plasma at the time of diagnosis and the level of tolerance. The available treatment consists of adopting a diet restricted in phenylalanine and daily consumption of supplements rich in amino acids, vitamins, and minerals, which should be maintained throughout the patient’s life [1, 2, 4].

Patients with PKU often experience cognitive impairments [5], as well as psychological and social impacts, due to the strict dietary restrictions required for treatment [6, 7]. These factors, along with social stigma and daily challenges, can lead to mental health impairments and a high incidence of psychiatric disorders such as anxiety and depression [8]. Caregivers of children with PKU also face significant burdens, impacting their financial, social, and psychological domains [9–11]. To assess the impact on quality of life, it is important to consider the multidimensional nature of the concept, including the physical, psychological, social, environmental, spiritual, and level of independence domains [12]. Quality of life assessment is an important factor in medical decision-making, as it can indicate little benefit from therapy and predict treatment success [13]. There are diverse instruments available to assess quality of life, including self-report and proxy report measures, single indicator and profile approaches, and generic and condition-specific populations [14].

However, despite its relevance, little is known about the QoL of phenylketonuria patients, their caregivers’ QoL, and the variables that influence it. To the best of our knowledge, only two literature reviews have been published in this context, each with a different target population: one article included only studies focused on the quality of life of adults with PKU [15] (including nine studies), whereas the other assessed only the QoL of caregivers of children or adolescents with PKU [16] (including eight studies). Therefore, this scoping review presents an updated overview of the literature concerning the entire PKU population.

This review aimed to identify, characterize, map, and summarize existing knowledge about the quality of life of PKU patients and their primary caregivers. In addition, this study aimed to provide an overview of this population’s current QoL and identify the impact factors associated with better or worse outcomes in the different domains of QoL and QoL in general. The following research questions guided the review:

1. What evidence is available about the quality of life of PKU patients and their primary caregivers?
2. What are the main characteristics (e.g., authors involved, period and place of publication, country, study design, population assessed) of the studies, methods, and instruments used to access QoL in this population?
3. What factors are significantly associated with the QoL of patients and their caregivers? Which factors are sources of improvement or impairment of the QoL of patients?

## Methodology

We conducted a scoping review of the methodological framework proposed by Arksey and O’Malley [17] (2005) and later improved upon that of Levac et al. [18] (2010) and Peters et al. [19] (2020). A scoping review is characterized by identifying, mapping, and summarizing the knowledge on a topic of interest and indicating aspects yet to be addressed [20–22]. Although this method has an exploratory approach, a scoping review also presents some characteristics of a systematic review, such as protocol and transparency, systematization, and replicability in its data search and extraction processes [19, 20, 23]. This scoping review is part of a larger project that is focused on the impact of phenylketonuria and its treatment in the lives of patients and their caregivers (Project PSIKUS – Impact of Phenylketonuria (PKU) and its Treatment in the Lives of Patients and Their Caregivers. Research and Ethics Committee number CAAE: 62035822.5.0000.5327).

### Protocol

Our protocol was drafted using the Preferred Reporting Items Checklist for Systematic Reviews and Meta-Analyses Extension for Scoping Reviews (PRISMA-ScR) [24]. The scoping review protocol was registered with the Open Science Framework on 8 May 2023 (available at https://osf.io/cmauq/).

### Literature search strategy

To develop the search strategy, librarian assistance with experience in literature review was consulted, and descriptors were defined by using the controlled vocabularies MeSH, Emtree, and Decs. Those terms were combined via AND and OR Boolean operators. English and Portuguese vocabulary was used. The relevant studies were identified by searching the electronic databases PubMed, PsycINFO, EMBASE, Scopus, the Cumulative Index to Nursing and Allied Health Literature (CINAHL), and the Virtual Health Library (BVS). The final words were divided into two categories: (1) diagnostic terms and (2) psychosocial terms.

”phenylketonurias” OR “phenylketonuria*” OR “PKU” OR “phenylketonuric*“.
”Treatment Adherence and Compliance” OR “therapeutic adherence and compliance” OR “treatment adherence” OR “therapeutic adherence” OR “adherence” OR “psycholog*” OR “psychosocial factors” OR “psychological factor” OR “Quality of life” OR “life style” OR “life quality” OR “health-related quality of life” OR “HRQOL” OR “Mental Health” OR “Social Interaction” OR “Psychosocial Support Systems” OR “social support” OR “social aspects” OR “social network” OR “Social Behavior” OR “social connectedness” OR “social acceptance” OR “belonging” OR “reference group” OR “self-efficacy” OR “self-management” OR “patient activation” OR “self-care” OR “self-confidence” OR “self-esteem” OR “adaptation, psychological” OR “behavior adaptive” OR “behavior change” OR “change strateg*” OR “lifestyle changes” OR “life changes” OR “coping” OR “coping behavior” OR “coping skill*” OR “coping strateg*” OR “resilience, psychological” OR “psychological resilience” OR “emotional adjustment” OR “expectations” OR “anxiety” OR “stress, psychological” OR “psychological stress” OR “Social Discrimination” OR “Social Stigma” OR “anxiety social” OR “social anxiety” OR “Social Isolation” OR “Peer Pressure” OR “Need for Approval” OR “Social Approval” OR “Social Reinforcement” OR “Caregiver Burden” OR “burden caregiver*” OR “burnout caregiver*” OR “caregiver exhaustion” OR “burden“.
(1) AND (2).

### Article selection

The inclusion criteria used in this scoping review were related to (a) the sample, that is, patients or caregivers of patients with PKU, and (b) the characteristics of the studies, including primary quantitative methodology studies published between January 2000 and February 2023 that address the experiences of patients or caregivers and psychosocial implications associated with living with PKU and its treatment. The exclusion criteria were as follows: studies that did not report psychosocial outcomes; studies that were written in a language other than English or Portuguese; studies that did not delineate the perspective of patients with PKU; and secondary research, literature reviews, protocols for ongoing studies and trials, case reports, or in vitro or animal studies.

The search results were imported into a bibliographic reference management tool (*Zotero*) to manually remove reference duplicates. The material was then transferred to another software (*Rayyan*) [25] to proceed with the two levels of screening: a title and abstract evaluation followed by a full-text review. The software *Rayyan* (https://www.rayyan.ai/) provides independent, blinded, and collaborative analysis tools. Abstracts that did not present enough information for a decision were advanced to the next step for a full reading. References that did not meet the inclusion criteria or were considered by both reviewers as not relevant for this study were excluded from the next step. The studies available were accessed and read by two reviewers independently. They selected those for the final review material on the basis of the inclusion and exclusion criteria, and conflicts were debated and resolved in consultation with a third author. The reasons for excluding articles have been identified and listed. The entire article selection process was recorded using a PRISMA-ScR flowchart [24].

### Charting the data

For the next stage, we developed a data extraction framework to map study characteristics on the basis of discussion among the authors and the Strengthening the Reporting of Observational Studies in Epidemiology (STROBE) checklist [26]. The main categories were author(s), article title, year, country of origin of data, characteristics of participants (sex, age, PKU classification, and treatment), sample size, methodology (including measured variables and instruments used), and main findings. We also extracted information on statistically significant effects on QoL (and specific domains) according to the significance level values established by the authors of the articles [reported in the supplementary material]. Before the review process, the authors piloted the data extraction framework to evaluate whether it was consistent and coherent with the study’s goals. Using Microsoft Excel 2010, two authors independently included data from the final studies selected for review. When necessary, a third reviewer was consulted to resolve potential disagreements.

## Results

**Fig. 1:**
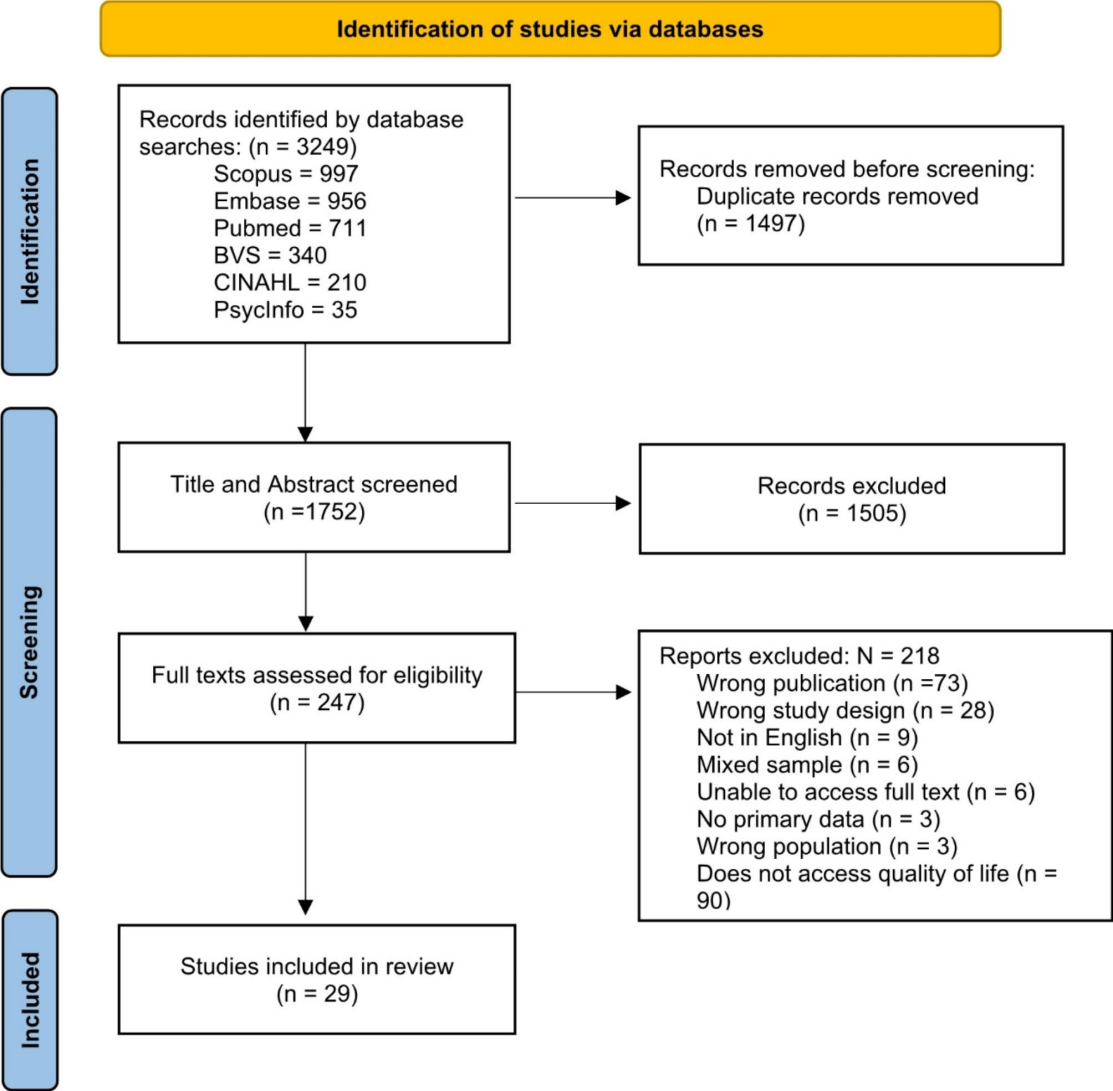
PRISMA flowchart of the selection process of included studies

The search resulted in 3.249 records. After removing duplicates, it was reduced to 1.752. Next, titles and abstracts were screened, and 1.505 were excluded for failing to meet the inclusion criteria. In the next stage, after the full-text screening, 218 articles were excluded. Twenty-nine articles were included in the final review. The selection process is illustrated in Fig. 1.

Among the 29 studies included for analysis in the present scoping review, 13 countries, 1.472 phenylketonuric patients, and 1.192 caregivers were included. Twenty-six studies were cross-sectional [8, 27–51], and three longitudinal studies [52–54] lasted four months to one year. The countries with the highest number of studies were Germany (*n* = 6), the Netherlands (*n* = 4), Iran (*n* = 4), the United Kingdom (*n* = 2), Italy (*n* = 2), Greece (*n* = 2) and Australia (*n* = 2). Studies were also conducted in Brazil, Hungary, Poland, Spain, Tunisia, and Turkey, with one occurrence each. European countries accounted for 65.5% (*n* = 19) of the publications. Three studies were multicenter studies [28, 34, 41]. The number of publications ranged from 2002 to 2022, with the highest concentration occurring from 2011 to 2021 (*n* = 24).

Eighteen out of 29 included studies (62%) assessed the quality of life of patients with PKU exclusively; in 10 (34,4%) studies, only the quality of life of caregivers of PKU patients was evaluated; and in one study, both the patient and the caregiver were assessed [28]. In all studies whose samples included adult participants (patients or caregivers), self-reported measures were used to assess quality of life. On the other hand, in the studies that evaluated the quality of life of pediatric patients, most used both self-report and proxy report measures (i.e., when caregivers are used as informants) [27, 28, 33, 36, 40, 42, 49]. In cases where the patient was not old enough to answer the self-report questionnaires, the parent version was used as the only measure of the child’s QoL [44]. The quality of life of children or adolescents was evaluated only by self-reports in three studies [29, 32, 50] and exclusively by proxy reports in one study [44]. In three studies, control groups were used, consisting of relatives not affected by PKU, acquaintances/friends, and/or unrelated healthy individuals. For summary information on the included studies on patients’ quality of life, please refer to Table 1, and for information on caregivers’ quality of life, please consult Table 2.

**Table 1:** Data charting of the included studies with phenylketonuria patients

Authors, year, country	Research design	Sample size (Mean age in years) % Sex	Control group	PKU type Diagnosis*	Medical treatment	Instrument	Main results
Aitkenhead et al., 2021 [8], UK	Quantitative, cross-sectional	149 adult patients (32.38 ± 9.04) 55% females	74 patients’ healthy relative	NR All early-treated	49% classified themselves as on dietary treatment, 30% as off-treatment, and 11.6% as partially adherent (restricted Phe or supplementation)	SF-36	The authors detected no significant differences in QoL between PKU patients and the control group. They identified QoL differences between self-report dietary adherence subgroups: partially-adherent patients obtained lower scores on physical and mental QoL when compared to totally adherent and non-adherent groups.
Alptekin et al., 2018 [50], Turkey	Quantitative, cross-sectional	63 children to adult patients (15.7 ± 6.4) 65.1% females	N/A	NR According to the authors, they invited individuals diagnosed with PKU without regard to any criteria.	Of all patients treated with AAM supplementation, 95.3% were combined with a restricted diet, and 17.4% were combined with pharmacological treatment (BH4).	PKU-QOL	Children reported worse scores in most domains, considering the four modules of the PKU-QoL, compared to adolescents and adults with PKU. On the other hand, some domains seem to affect the three groups with the same intensity, such as “tiredness,” “emotional,” and “social impact of PKU,” “anxiety-blood Phe levels,” “taste-supplements,” and some domains related to the dietary protein restriction module.
Barta et al., 2020 [35], Hungary	Quantitative, cross-sectional	88 adult patients (median age = 31, IQR = 25–40) 52% females	N/A	75% Classical; 25% non-classical All early-treated	Phe restricted diet with supplementation of AAM	PKU-QOL	Most patients rated their overall health status as “good” or better. Domains scores indicating moderate impact were: “Tiredness,” “Anxiety – Phe levels during pregnancy,” “Emotional impact of PKU,” “Taste – supplements,” “Guilt if poor adherence to supplements,” and “Guilt if poor dietary protein restriction not followed.” No domain had scores indicating a severe impact. In patients with Classical PKU, all QoL domains were positively correlated with Phe levels, indicating that patients with good metabolic control in the short or long term had a lower impact on QoL. Patients with classical PKU reported a larger financial burden impact than Non-Classical patients.
Bik-Multanowski et al. 2008 [52], Poland	Quantitative, prospective trial	53 adult patients (24.0) 53% males	N/A	Classical All early-treated	Patients resumed a strict low-phenylalanine diet after enrolment in the program.	Psychological General Well-Being Index	Before reintroducing the restricted diet, positive well-being was observed in 55% of participants. Twenty-nine patients managed to maintain the diet for at least three months, and only ten continued in the study for up to nine months. The authors observed improvement in QoL after three months in most participants with severe or moderate baseline discomfort.
Bosch et al., 2007 [48], Netherlands	Quantitative, cross-sectional	32 adult patients (24.6 ± 2.6) 69% females	N/A	NR All early-treated	Phe restricted diet with supplementation of AAM	RAND-36 Health Survey The cognitive scale of the TAAQOL	The authors found no differences between PKU patients and normative data.
Bosch et al., 2015 [28], multicenter	Quantitative, cross-sectional.	306 children to adult patients (NR) 54% females	N/A	68% Classical; 32% Mild NR	Phe restricted diet and/or supplementation of AAM and/or pharmacological treatment (BH4)	CHQ-PF 28 PedsQL PKU-QOL SF-36	The scores of the generic measures of QoL in children and adolescents were similar to the general population; considering the scores of adults with PKU, the physical domains were higher, and the mental domains were lower when compared to the normative data. Considering the scores of the PKU-specific questionnaire, patients with more severe protein restriction reported a greater impact of the disease on daily life, and BH4 treatment was associated with better scores in all ages.
Cazzorla et al., 2014 [40], Italy	Quantitative, cross-sectional	43 children to adult patients (17.1 ± 9.0) 53% female Caregivers (of 6–16 years old patients, *proxy report*).	N/A	51% Mild; 49% Classical All early-treated	Classic PKU: Phe restricted diet. Mild PKU: 36% treated with BH4 and low protein foods, 36% with BH4 and Phe restricted diet and AAM, and 27% treated only with BH4	PedsQL WHOQOL-100	The authors found no significant differences in QOL between PKU patients (or parent reports) and normative data. Compared to patients with classic PKU, higher QOL scores were observed in patients with mild PKU on BH4 treatment. Compared to employed or unemployed adults, better QOL was observed in students and adults with higher education.
Cotugno et al., 2011 [27], Italy	Quantitative, cross-sectional	41 patients and their caregivers – *self* and *proxy report* (10.7 ± 6) 61% males	N/A	73% Classical; 27% Mild All early-treated	Low Phe diet, Phe-free protein substitutes, long-chain polyunsaturated fatty acids and vitamins.	CHQ CHQ-PF 50 SF-36	The study found that children and adolescents with PKU had significantly lower QoL scores than healthy populations, particularly in the physical and psychological summary scores. There was a significant correlation between adherence to the prescribed diet and QoL in specific domains, such as Global Health and Family Activities. Adolescents and young adults showed a different pattern, with generally better QoL scores than younger children, although adherence to diet was consistent across age groups. The study highlighted that children of mothers with higher education levels tended to have better adherence and slightly better QoL outcomes.
Das et al., 2013 [29], Germany	Quantitative, cross-sectional	51 adult/adolescent patients (26.6 ± 6.6) 62.7% females	N/A	100% Classical All early-treated	Four dietary groups: 42% of patients followed a protein-restricted diet supplemented with amino acid mixtures (AAM) [PKU recommended], 8% of patients followed a vegan diet supplemented with AAM; 14% of patients followed a vegan diet without taking AAM; 36% of patients reported eating a normal diet (Presumed normal eating habits).	Alltagsleben (AL) questionnaire	No differences were found between PKU patients and the general population in the scores of the subscales of the QoL assessment. No significant differences were found in quality of life or mood among the four diet conditions, indicating that patients who followed strict dietary recommendations did not feel worse than those with a more relaxed diet.
Demirdas et al., 2013 [33], Netherlands	Quantitative, cross-sectional	69 children to adult patients (18.4 ± 10.2) 52% females Mothers – *proxy report* (patients 4–7 years of age)	N/A	NR All early-treated	Phe restricted diet with supplementation of AAM	PedsQL TAAQOL DISABKIDS chronic generic module.	Children and adolescents with PKU reported higher HRQoL scores than the general population, and adults reported similar scores. PKU Children showed no differences in HRQoL scores before and after the administration of the BH4.
Gassió et al., 2003 [30], Spain	Quantitative, cross-sectional	15 patients (27.5) 66% females	N/A	100% Classical 53.3% late diagnosis	Patients resumed treatment after a period of dietary discontinuation or initiated the restricted diet after a late diagnosis.	Ad hoc questionnaire	60% of patients felt that their quality of life improved after resuming or starting a phenylalanine-restricted diet compared to when they were off the diet. 53% of patients reported feeling calmer, quieter, and less easily upset, while 40% noted being more alert and better able to maintain attention while on the diet. 33% of patients felt happier, and 27% reported feeling more energetic and vital. 20% of patients felt less impulsive and aggressive and noted they were less argumentative than before diet resumption. 53% of patients described their health as very good and 47% as good, with 40% reporting that their current health on the diet was better than their health when off the diet.
Hatami et al., 2015 [51], Iran	Quantitative, cross-sectional	82 patients (41% were younger than 20 years old; 49% were between 20–29 years old; 10% were 30 years or older) 58% females	N/A	NR All late diagnosis	NR	WHOQOL-BREF	The patients were late-diagnosed phenylketonuria (PKU), generally experiencing a low quality of life. Patients over 40 years old had significantly lower mental health compared to other age groups. The patient’s educational level notably influenced the quality of life, with those receiving higher education experiencing a better quality of life. There were no significant differences in QOL between male and female patients. The social and environmental aspects of QOL were particularly low among the patients.
Hujibregts et al., 2018 [49], Netherlands	Quantitative, cross-sectional	90 children to adult patients (21 ± 10.1) 58.8% females	109 healthy controls	NR All early-treated	Dietary treatment: 30% receive BH4 treatment.	TAAQOL TACQOL-PF TACQOL-CF	Except for the lack of autonomy reported by parents, children with PKU showed an HRQL similar to the control group. Among adults with PKU, more differences were found compared to the control group: patients showed lower scores regarding cognition, depressive moods, and anger. Negative correlations were found between QoL domains and lifetime and concurrent Phe levels.
Klimek et al., 2020 [34], Germany	Quantitative, cross-sectional.	144 adult patients (33 ± 8.5) 66% females	N/A	NR All early-treated	82% Phe restricted diet 84% Amino acid moisture 18% not keep any dietary regimen 10% neve take AAM	Ad hoc questionnaire	91% of early-treated adult PKU patients considered their quality of life good, and 83% rated their health as at least good. 70% of the patients reported feeling better when adhering to a diet than when they did not. Despite the strict dietary regimen, most patients felt the diet’s restrictions on their daily life were low.
Landolt et al., 2002 [44], Germany	Quantitative, cross-sectional	37 pediatric patients and their caregivers - *proxy report* (10.9 ± 9.9) 51.8% males	N/A	NR All early-treated	Phe restricted diet with supplementation of AAM	TACQOL-PF	Only in the “Positive Emotion” domain, PKU patients showed lower scores compared to healthy children, according to parent-report. QOL was associated with Phe level during the first year of life.
Palermo et al., 2020 [31], UK	Quantitative, cross-sectional	36 adult patients (27.4 ± 8.3) 75% females	40 healthy people	100% Classical All early-treated	Six patients were on an unrestricted diet, and 30 were on a low-protein diet.	SF-36	No differences were found in mental and physical health-related QoL scales between patients with PKU and the control group. Better cognitive functioning was associated with better physical QoL, and better physical QoL was associated with lower Phe levels and reduced Phe fluctuations across life.
Simon et al., 2008 [32], Germany	Quantitative, cross-sectional	67 adolescent or adult patients (median age = 25) 66% females	N/A	100% Classical All early-treated	NR	Profile of Quality of Life in the Chronically Ill Questionnaire	No differences in QoL scores were found between patients and the control group. Women with PKU reported significantly lower levels of positive mood when compared to men with PKU.
Thimm et al., 2013 [36], Germany	Quantitative, cross-sectional	50 pediatric patients and their caregivers – *self and proxy report* (Median age = 9.9) 64% females	N/A	NR All early-treated	Phe restricted diet with supplementation of AAM	KINDL-R questionnaire	No differences in the total self- and parent-reported QoL score were found between PKU patients and healthy peers. Only the everyday functioning dimension showed a difference in parent-reported QoL, and parents rated PKU patients lower. A positive correlation between everyday functioning dimension and metabolic control was found.
Vieira Neto et al., 2018 [42], Brazil	Quantitative, cross-sectional	51 pediatric patients and their caregivers - *self and proxy report* (NR) 56.8% males	N/A	64% Classical; 33% mild or moderate; 3% no information All early-treated	Phe restricted diet with supplementation of AAM	PedsQL	The self-reports of pediatric PKU patients’ QoL and parent reports were lower compared to a normative sample. A correlation between treatment adherence and quality of life was not observed.

Notes: *CHQ* = Child Health Questionnaire; *CHQ-PF 28* = Child Health Questionnaire 28 items - Parent Form; *CHQ-PF 50* = Child Health Questionnaire 50 items - Parent form; *PedsQL* = Pediatric Quality of Life Inventory; *PKU-QOL* = Phenylketonuria - Quality of Life questionnaire; *TAAQOL* = TNO-AZL Adult Quality of Life questionnaire; *TACQOL-CF* = TNO-AZL Child Quality of Life Questionnaire - Child form; *TACQOL-PF* = TNO-AZL Child Quality of Life Questionnaire - Parent form; *SF-36* = 36-Item Short Form Health Survey; *WHOQOL-100* = World Health Organization Quality of Life − 100; *WHOQOL-BREF* = World Health Organization Quality of Life 26 items

AAM = amino acid mixture

N/A = Not applicable

NR = Not reported

*Early or late diagnosis as informed by the authors

**Table 2:** Data extraction of the included studies with caregivers/parents of patients with PKU

Authors, year, Country	Research design	Sample size (mean age in years) % Sex	Patient’s mean age in years. %Sex	Control group	Patient’s PKU type Diagnosis*	Patient’s medical treatment	Instrument	Main results
Ben Abdelaziz et al., 2020 [43], Tunisia	Quantitative, cross-sectional	65 caregivers (No mean age reported) 62% female	8.9 ± 6.2 NR	N/A	NR Late diagnosis	Phe restricted diet	SF-36	Income and sex were factors associated with worse QoL, i.e., being female and living with less than one minimum wage. Higher education was associated with better QoL, and fathers showed higher QoL than mothers. Parenting an autistic child was also associated with worse QoL levels.
Bosch et al., 2015 [28], multicenter	Quantitative, cross-sectional	253 caregivers (41.6 ± 6.5) 72% female	NR 54% females.	N/A	68% Classical; 32% Mild NR	Phe restricted diet and/or supplementation of AAM and/or pharmacological therapy (BH4)	CHQ-PF 28 PKU-QOL	Parents’ QoL scores were comparable to normative data, except for a lower score on the emotional parental impact domain, indicating a higher emotional impact than the general population.
Etemad et al., 2020 [37], Iran	Quantitative, cross-sectional	240 caregivers (36.8 ± 7.8) 55% female	8.7 ± 8.1 55% male	N/A	NR 52.8% early-treated	NR	WHOQOL-BREF	Parents of children with PKU reported low levels of QoL. Demographic factors associated with two or more QoL domains: parents’ age, parents’ education, child’s age, and household income.
Fidika et al., 2013 [41], Germany	Quantitative, cross-sectional.	89 caregivers (39 ± 6.9) 85% female	9 ± 5.2 51.7% female	N/A	NR All early-treated	NR	The Ulm Quality of life Inventory for Parents of chronically ill children	Most parents reported a positive appraisal of their quality of life. Lower QoL was found in parents of preschool-aged children compared to parents of school-aged children and adolescents. Age of children and parents, perceived social support, parental coping, and family stress were predictors of QoL.
Iakovou and Schulpis 2019 [53], Greece	Quantitative, intervention	42 caregivers (26.8) 100% female	NR	N/A	NR NR	NR	Ad hoc questionnaire	Mothers of children with PKU and those with a college education were the most likely to benefit from psychological support compared to the primary and high school groups.
Iakovou et al., 2019 [47], Greece	Quantitative, cross-sectional	110 caregivers (25.7) 100% female	NR	N/A	NR NR	NR	Ad hoc questionnaire	Mothers who completed only elementary school and resided in a city with higher population density reported higher levels of damage to QoL. In addition, mothers with a university degree reported the lowest level of social discrimination, and those who resided in a small town reported the highest levels.
Irannejad et al., 2018 [46], Iran	Quantitative, cross-sectional	124 caregivers (39.6 ± 10.6) 50% female	64.1% were younger than ten years old. 57.6% male	N/A	NR 78.8% Late diagnosis	NR	SF-36	Parents of children with PKU reported lower levels of QoL compared to average levels. A negative association was found between stress level and QoL.
Mahmoudi-Gharaei et al., 2011 [45], Iran	Quantitative, cross-sectional	49 caregivers (35.6 ± 8.8) 59.2% female	9.8 ± 6.6. 51% female	N/A	NR The mean age of PKU diagnosis was 4.7 years ± 3.7	NR	WHOQOL-BREF	Compared to the general population, lower levels of quality of life were found in caregivers of children with PKU. Not being employed and levels of depression and anxiety were factors associated with these parents’ quality of life.
Mitchell et al. 2021 [54], Australia	Quantitative, longitudinal, non-randomized trial	17 caregivers (37.7 ± 5.4) 100% female	6.2 ± 3.2 58.8% female	N/A	47% Classical NR	Phe restricted diet with supplementation of AAM	PKU-QOL	No significant differences were observed in caregivers’ QoL scores after the intervention.
Morawska et al., 2020 [39], Australia	Quantitative, cross-sectional	18 caregivers (38.6 ± 5.9) 100% female	6.89 ± 3.68 66.7% female	N/A	44.4% Classical; 27.8% Mild; 27.8% Hiper-Phe All early-treated	Phe restricted diet with supplementation of AAM	PKU-QOL	The highest reported impact was related to parental guilt due to treatment adherence levels and perceived anxiety in the child when performing the blood test. Several factors related to the mother and child were associated with QoL domains, such as emotional maladjustment, behavioral difficulties, parental stress, dysfunctional parent-child interaction, challenging child, and hyperactivity. Higher lifetime phe levels also showed an association with some domains of QoL.
ten Hoedt et al., 2011 [38], Netherlands	Quantitative, cross-sectional	116 caregivers (40.7 ± 6.3) 56.9% female	8.7 ± 5.0 NR	69 caregivers of children with galactosemia; 108 caregivers of children with other metabolic disorders 430 caregivers of healthy children	NR / NR	NR	TAAQOL	Parents of healthy children, children with PKU, or children with galactosemia showed no differences in QoL. In addition, parents of children with PKU reported better QoL than those with other metabolic diseases. In parents of children with PKU, having older children and more emotional support were associated with better HRQoL, whereas loss of friendship was associated with worse HRQoL.

Notes: *CHQ-PF 28* = Child Health Questionnaire 28 items - Parent Form; *PKU-QOL* = Phenylketonuria - Quality of Life questionnaire; *TAAQOL* = TNO-AZL Adult Quality of Life questionnaire; *SF-36* = 36-Item Short Form Health Survey; *WHOQOL-BREF* = World Health Organization Quality of Life 26 items

AAM = Amino acid mixture

N/A = Not applicable

NR = Not reported

*Early or late diagnosis, as informed by the authors

### Characteristics of the participants

Seven studies (7 out of 19) [8, 31, 34, 35, 48, 51, 52] included only adult patients (≥ 18 years old), whereas in the other six studies [27, 28, 33, 40, 49, 50], in addition to adults, the sample also included children (above three years old) and adolescents. The remaining studies evaluated adolescents and adults (≥ 16 years; 15.7%, *n* = 3) [29, 30, 32] or pediatric patients only (ages ranging from 0 to 18 years; 15.7%) [36, 42, 44]. There was a predominance of female patients in the studies reviewed: in 84% of the studies (*n* = 16), the number of women/girls was greater than the number of men/boys. The sample size ranged from 15 to 306 (Md = 53). Eleven studies assessed the self-reported quality of life of parents and/or caregivers of patients with PKU. Among parents, the majority of participants were mothers, and four studies included only female caregivers [39, 47, 53, 54]. The number of caregivers ranged from 18 to 253 (Md = 99.5)^1^.

## Measurement tools

Different assessment tools were used in the studies to assess the QoL of patients with PKU and their parents. The most common ones are listed in Table 3.

**Table 3:** Most frequently used tools in quality-of-life assessment

Instrument	Type of measure (generic vs. disease-specific)	Studies with Patients	Studies with Parents/caregivers
Phenylketonuria - Quality of Life (PKU-QoL)	disease-specific	28, 35, 50	28, 39, 54
Medical Outcomes Short-Form Health Survey (SF-36)	generic	8, 27, 28, 31	43, 46
Pediatric Quality of Life Inventory (PedsQL)	generic	28, 33, 40, 42	-
TNO-AZL Questionnaire for Adult’s Health-Related Quality of Life (TAAQOL)	generic	33, 48, 49	38
The World Health Organization Quality of Life Questionnaire (WHOQOL-100 and WHOQOL-BREF)	generic	40, 51	37, 45
Child Health Questionnaire - Children and Parent Form (CHQ; CHQ-PF 50)	generic	27	27, 28
TNO-AZL Questionnaires for Children’s Health-Related Quality of Life - Children and Parent Form (TACQOL-CF; TACQOL-CP)	generic	44, 49	-

Two studies with patients [30, 34] and two studies with parents [47, 53] applied questionnaires developed especially for their research. Readers can find the names of the instruments mentioned in only one study in Tables 1 and 2.

### Quality of life of pediatric patients with PKU

The QoL of pediatric patients (< 18 years) was assessed in 12 studies, and 10 adopted generic questionnaires that compared data obtained with the general population and/or normative of the instrument. Significant differences were found in 60% of the studies (*n* = 6/10), and in five of these six investigations, PKU patients had worse scores than did the general population in one or more domains of QoL [27, 36, 42, 44, 49]. One article reported better QoL than did the general population: adolescents had higher scores on the psychosocial functioning domain and total score, whereas children had higher scores on physical functioning [33].

The QoL dimensions associated with a more significant impact on PKU pediatric patients were those related to academic functioning [33, 36, 42], emotional functioning [27, 42, 44], social functioning [27, 42], and physical health [27, 42]. Impairments in cognitive functioning (in adolescents only) [49], family functioning [27], and autonomy (in children only) [49] domains were also reported but with less frequency.

In two studies [28, 50], specific questionnaires to evaluate the QoL of patients were used, i.e., the PKU-QoL, and children had higher scores on all PKU-QoL modules, which indicates a greater negative impact of disease in all age groups. The most frequent symptoms among pediatric patients were slow thinking, a lack of concentration, and tiredness. The higher median score on the “PKU in general” module for children and adolescents was associated with anxiety about blood Phe levels. On the “Phe-free amino acid supplement administration” module, children and adolescents scored higher guilt in cases of poor adherence to supplements than did adults. Additionally, one of the highest scores in this module was related to the impact of the taste of these products, indicating that they dislike the taste of Phe-free amino acid supplements. The median scores in the “Dietary protein restriction” module were higher for items associated with guilt if protein restriction was not followed [28, 50]. A summary table is presented detailing the information on the quality of life of children’s patients with PKU as an additional file (see supplementary material Table S2).

### Quality of life of adult patients with PKU

Fourteen studies included adult patients in the sample. Of these, 10 used generic questionnaires to assess QoL and compared the results with normative scores, control groups, and/or healthy sample data. Most studies (7 out of 10) did not find statistically significant differences; i.e., in these studies, the quality of life of adults with PKU was similar to that of healthy individuals [8, 28, 29, 31, 32, 40, 48]. In the remaining studies (3 out of 10), impairments were observed in the psychological dimension of quality of life, with significantly lower scores than those of the general population in the domains of cognitive functioning [33, 49], depressive emotions, aggressiveness [49], vitality, social functioning, role-emotional limitations, and mental health [28]. One study reported significant differences in the physical dimension of quality of life. Nevertheless, in this case, phenylketonuric adults had higher scores in physical functioning, role-physical limitations, bodily pain, and general health domains [28].

On a specific questionnaire developed for patients with PKU, i.e., the PKU-QoL, tiredness [28, 50], and moodiness [50] were the most frequent symptoms among adults. In the “PKU in general” module, the strongest impact was from anxiety due to blood Phe levels [50] and the emotional impact of PKU [28]. In female patients, anxiety about blood Phe levels during pregnancy had the highest median scores of all domains [28, 50]. In the “Phe-free amino acid supplement administration” module, the taste of supplements [28, 50] and guilt associated with poor adherence to supplements [28] had the highest median scores, indicating that adult patients perceived these products as unpleasant. Guilt if dietary protein restriction was not followed [28, 50] and food enjoyment [50] were the most negatively impacted domains of the “Dietary protein restriction” module. A summary table is presented as an additional file detailing the quality of life of adult patients with PKU (see supplementary material Table S1).

### Quality of life of parents/caregivers of patients with PKU

Among the six studies that adopted generic measures to assess QoL in parents, most (*n* = 5) identified impairments in at least one QoL domain or total QoL domain, with significantly lower scores than the normative data and/or the control group [37, 38, 43, 45, 46]. A study [43] with a Tunisian population reported that PKU parents’ mental and physical health summary scores were comparable to those of the Tunisian general population. Another study [38] revealed no differences in QoL between caregivers of children with PKU and parents of healthy children or parents of patients with galactosemia; in the same study, caregivers of children with PKU had better scores than caregivers of patients with other metabolic disorders in most domains [38]. In a study that did not compare parents’ QoL with that of other groups, caregivers rated it as good, with the highest scores in the “satisfaction with family” domain and the lowest in the “self-development” domain [41].

On the other hand, few studies have reported impairments in QoL dimensions in caregivers via generic measures [45, 46]. Among the studies that described domains individually, those with scores significantly below the average were emotional/psychological [28, 37, 45], social, environmental [37, 45], and physical [45]. Within the studies using a PKU-specific questionnaire, parents reported higher median scores, indicating greater impact on the following domains: guilt if poor adherence to supplements, guilt if dietary protein restriction is not followed, anxiety about blood Phe levels of children or adolescents, and the impact of the child’s anxiety during blood tests [28, 39]. A summary table is presented detailing the information on the quality of life of parents as an additional file (see Table S3).

### Factors associated with QoL among PKU patients and caregivers

Sociodemographic (i.e., sex, education, income, and age), psychosocial (i.e., neuropsychological aspects, mental health, social support, and attitudes toward diet), treatment adherence, and disease-specific (i.e., disease severity and tolerance to Phe intake) factors were associated with better or worse QoL in the PKU population. A summary of the relevant variables is presented in Table 4.

**Table 4:** Summary of factors associated with QoL of patients and caregivers

	Factor	Association with patients’ QoL	Association with caregivers’ QoL
Sociodemographic	Sex	X	X
	Age	X	X
	Educational level	X	X
	Employment		X
	Household income		X
	Number of children per family		X
Psychosocial	Cognitive functioning	X	
	Patients’ behavioral aspects		X
	Mental health		X
	Social and emotional support		X
	Parenting aspects		X
	Attitudes toward diet	X	
Adherence to treatment	Treatment adherence	X	
Disease-related	Disease severity/ Phe tolerance	X	X
	Treatment duration	X	
	Metabolic control	X	
	Lifetime Phe levels	X	X
	Phe levels on last ten years	X	
	Phe levels during the first year of life	X	
	Concurrent Phe level	X	

### Sociodemographic factors and QoL

#### Sex, education, income

In three studies, female patients had the worst scores on QoL domains [32, 42, 49], but one study reported that male patients reported greater damage to QoL than female did [40]. Educational level was also positively associated with QoL in PKU patients, i.e., those with the highest educational level had better QoL scores [40, 51]. Among caregivers, factors such as female sex, low education [37, 43, 47], and low household income [37, 43] were associated with the worst quality of life. Middle-income pediatric patients evaluated emotional functioning dimensions better than high-income pediatric patients did [42]. Caregiver occupation was significantly associated with the physical, social, and environmental domains of QoL [45]. The number of children per family (with or without a PKU diagnosis) was negatively correlated with physical health dimensions [43].

#### Age

In patient group comparisons (children, adolescents, and adults), children were significantly more impacted by PKU than adolescents and adults were, except in the “Dietary protein restriction” module [50]. Adolescents mentioned a greater impact in domains associated with family functioning (“Family cohesion” and “Parental impact–time”) [27]. Compared with younger adults, adults above 40 years of age had poorer mental QoL [51]. The caregiver’s age and child’s age under their care also influenced QoL: the data seemed to converge on a positive association, meaning that older caregivers had better QoL [37, 41]. With respect to child age as a factor for parents’ QoL, in one study, parents of older children had lower QoL on some dimensions and in terms of psychological health [37], but in another study, an older age of the child was associated with a better mental QoL [38]. Accordingly, in another study, parents of children under six years reported the lowest total QoL scores, differing from parents of school children and parents of adolescents [41].

### Psychosocial factors and quality of life

#### Neuropsychological aspects

Patients’ cognitive functioning was associated with physical health: those with better performance on cognitive tasks (i.e., total IQ, complex executive functions, visuospatial attention speed, visuomotor coordination, short-term memory, sustained attention, spoken language, verbal learning and memory, visual learning and memory, language speed and orthographic process accuracy) had better QoL in the physical health domain [31]. Caring for a child with PKU and autism was associated with the worst global, physical, and mental parental QoL [43]. Additionally, children’s agitation and mental disability were associated with lower parental QoL scores on some dimensions [43].

#### Mental health

Lower scores for the QoL of caregivers were associated with greater perceived stress [39, 41, 45, 46]. Family stress predicted parents’ QoL [41] and the social impact of PKU [39]. Anxiety and depression in caregivers were negatively associated with most of their QoL dimensions, including physical and psychological health, social relationships, and environmental health [45]. Parents who described a greater intensity of child behavioral difficulties and emotional maladjustment reported a greater frequency of PKU symptoms and showed a greater emotional impact of PKU [39].

#### Social Support

Perceived social and emotional support was associated with higher QoL in caregivers [38, 41]. Similarly, loss of friendship was negatively associated with mental QoL, and emotional support was shown to mediate the relationship between perceived stress due to PKU management and parental QoL [38].

#### Attitudes toward diet

Patients who perceived dietary compliance as easy had higher mental health scores associated with QoL [31].

### Treatment adherence and quality of life

#### Studies with adult patient

Current levels of adherence were significantly associated with several domains related to QoL. According to the disease-specific questionnaire, patients with better actual adherence had lower scores for slow thinking, the impact of the taste of low-protein food, food temptation, the practical impact of supplements, the overall impact of dietary protein restriction, and the practical impact of dietary protein restriction [35]. Correlations were found between current adherence and the overall impact of PKU, adherence to dietary protein restriction, the emotional impact of PKU, anxiety due to Phe levels, and the social impact of dietary protein restriction, indicating that patients with good actual adherence had a lower impact on QoL [35]. For generic measures, high Phe levels were correlated with lower scores on the sexuality dimension [49].

High lifetime Phe levels were associated with lower scores on domains related to sleep, pain, sexuality, and anger [49]. Patients with long-term metabolic control achieved higher scores on trembling hands, stomach aches, and food enjoyment dimensions [35]. Phe values from the last ten years were positively correlated with the following QoL domains, as measured by the PKU-QoL: trembling hands, social impact of dietary protein restriction, adherence to supplements, adherence to dietary protein restriction, and the practical impact of PKU [35]. Fluctuations in Phe levels across life were associated with the worst physical health [31]. Partially adherent adults – who either took supplements or followed the restrictive diet –had lower mental and physical QoL scores than did those who were fully compliant or fully non-compliant [8]. A study with noncompliant adults with phenylketonuria after resumption of the diet reported significant improvement in QoL [52]. Similar findings were reported by Gassió et al. (2003) [30], who reported that 60% of patients reported improved QoL after diet initiation or reintroduction.

#### Studies with pediatric patients

The Phe concentration during the first year of life was inversely correlated with the cognitive and negative emotional dimensions of QoL [44]. Additionally, good metabolic control was associated with higher QoL in the everyday functioning domain, which comprises items related to academic functioning, as rated by parents of children and adolescents [36]. Time on treatment was an important variable that positively influenced QoL in pediatric patients [40]. Significant correlations were found between adherence and the scores of the Child Health Questionnaire’s Global Health and Family Activities scales, whereas only marginally significant correlations were found for Global Behavior. No correlation was found between adherence and the two summary measures (physical functioning and psychological) or between adherence and the single domains of the questionnaire [27].

### Disease severity and tolerance of phe intake

Studies with adult patients have reported that the impacts of Phe-free amino acid supplementation and dietary restriction are lower in adults receiving tetrahydrobiopterin (BH4) treatment than in those with no BH4 intake [28], and better scores on pain, social functioning, happiness, and anger domains have also been reported [49]. Compared with patients with less severe PKU, adult patients with classic PKU have a greater financial burden [35].

Studies with pediatric patients revealed that adolescents and parents of children with classic PKU reported poorer dietary adherence and a greater impact on practical daily life and emotional domains due to amino acid mixture supplementation and dietary restriction [28] and lower total QoL [42] than patients with mild or moderate PKU. In addition, children and adolescents receiving complementary pharmacological treatment with tetrahydrobiopterin (BH4), which allows higher tolerance to Phe intake, showed a less practical impact of amino acid mixture supplementation. Compared with patients with no BH4 intake treated with a restricted diet only, adolescents also had a lower impact of dietary restriction [28]. The QoL scores of patients with mild PKU treated with BH4 were higher than those of pediatric patients with classic PKU treated with diet [40].

A study [39] with parents of children with classic PKU reported a greater financial impact of the disease and higher scores on guilt due to poor adherence to dietary restrictions and Phe-free amino acid supplement intake than did caregivers of children with mild PKU and hyperphenylalaninemia. Elevated Phe lifetime levels were associated with increased child PKU symptoms, increased anxiety due to blood Phe levels and parental impact on the child’s anxiety about the blood test, increased financial impact, and increased impact of the management of dietary protein restriction [39].

### Interventional studies aimed at improving the quality of life of caregivers

Studies addressing interventions to improve QoL related to PKU disease are limited to caregivers. Two experimental studies involving caregivers of phenylketonuric patients were included in this review. The first study [53] analyzed the effect of psychological support on mothers of children with PKU who suffered from social discrimination and presented impairments in quality of life. After one year of weekly counseling, a greater response to psychological support was observed—with a reduced impact on QoL and social discrimination—in mothers with a higher educational level. The authors argued that a better understanding of the importance of dietary restriction, the professional and family environment, and greater cooperation during the sessions might be related to years of formal education, suggesting that future interventions should consider educational level as a relevant factor. Another study [54] tested a brief intervention with caregivers of children with PKU aiming to stimulate parental self-regulation and positive parenting practices in the voluntary participants of the intervention. Although the intervention did not significantly affect caregivers’ quality of life, a decrease in ineffective parenting practices was observed. In addition, an impact on the children of the participants was observed, such as improved metabolic control in patients whose Phe levels exceeded the recommended limits before the intervention, with a moderate effect size.

## Discussion

For many years, research conducted with the PKU population has focused on investigating the neurological and cognitive damage common among these patients. However, interest in exploring the quality-of-life impacts associated with the disease has been growing exponentially, with 82.7% of such studies with patients and their caregivers dating between 2011 and 2021.

Different ways of accessing QoL were used, with 14 instruments used in patient samples and six with caregivers. Four studies used ad hoc questionnaires. Most articles adopted generic questionnaires (69%); the generic instruments used varied across studies, making it difficult to summarize the results since the concepts evaluated are diverse, and some domains are not equivalent across the questionnaires.

Only five studies used an instrument developed for the PKU population and their caregivers. This may be related to the fact that the final version of the first disease-specific questionnaire (i.e., PKU-QoL) was only available after 2015. The PKU-QoL includes variations for different respondents (children, adolescents, adults, and parents); its structure comprises PKU symptoms; the emotional, social, and overall impact of the disease; and the impact of treatment, including questions regarding dietary protein restriction and the administration of Phe-free protein supplements [55]. Whereas generic tools access universally important constructs (e.g., physical functioning, social functioning, level of independence, and mental health), the PKU-QoL questionnaire addresses the specific conditions patients and caregivers face.

All studies with adult patients or caregivers collected the data through self-report questionnaires. In contrast, most studies with pediatric patients used both self-reported and proxy-reported measures. Notably, in these cases, the use of both forms of reporting is prioritized since health decisions made by parents are influenced by their perceptions of the child’s quality of life [14].

Most studies did not observe significant harm to the QoL of adult patients. This could be related to the rigidity of PKU treatment, which is lessened as the patient approaches adulthood, with more flexibility in the diet [28], or, on the other hand, may be the result of the use of questionnaires that are not sensitive to the specifics of this population, such as special diet management and supplement administration. Generic QoL questionnaires are not designed to assess the inherent characteristics of a specific condition and may not be able to detect small changes in the QoL of particular diseases [13]. A moderate negative impact of PKU and its treatment was identified among adults in studies that assessed QoL through a disease-specific measure of PKU-QoL [35].

Despite the heterogeneity of the quality-of-life dimensions that compose the generic assessment tools, some similarities could be observed: damage to the emotional/psychological [27, 28, 37, 42, 44, 49] and social [27, 37, 42] dimensions of QoL were described by patients with PKU in all age groups and their parents. The negative impact on social aspects of life is frequently mentioned by PKU patients in qualitative studies [6, 56, 57]. Vegni and cols [57]. named PKU a “social disease” since its manifestation is not visible until the moment of eating. Socializing with peers when they enter school and later in the work setting is a concern among patients, who report feelings such as anger, shame, and fear of stigmatization in food-related situations. Not telling acquaintances about the condition, eating only at home, not going to food-sharing events and even eating forbidden foods in social situations were strategies adopted by patients to avoid misconceptions and stigma [6, 56, 57]. In this sense, in addition to the possible harm of nonadherence, these patients may also experience social deprivation.

When a disease-specific questionnaire across four versions of the PKU-QoL (children, adolescents, adults, and caregivers) is considered, the aspects most affected by the different reporters include anxiety about blood Phe levels, guilt related to poor adherence to dietary restrictions, and Phe-free amino acid mixture supplement intake [28, 35, 50]. This highlights the particular challenges PKU patients and their parents face, the emotional impact associated with disease and treatment management, social issues, the experience of struggles regarding uncertainty about their health in the future, the need to think about diet [58] constantly, and frustrations with health care providers [59], all of which are corroborated by the qualitative literature.

Conversely, better scores for physical health associated with QoL were found. The authors suggest that PKU patients may value their physical health more highly because they know the consequences of the disease if treatment is not followed and that patients and their caregivers who participate in surveys tend to be younger than the reference group respondents [28, 43].

Many factors were reported to be associated with total QoL or its dimensions. Sociodemographic characteristics were related to the QoL of patients and their caregivers. Among patients and caregivers, females were associated with the worst QoL. In addition to addressing cultural challenges, PKU patients also have to address concerns regarding maternal PKU [32]; anxiety about blood Phe levels during pregnancy achieved the higher median scores of all domains [28, 50]. Compared with male patients, female patients often report more emotional problems with PKU [60]. The lower quality of life for mothers seems to reflect that mothers are commonly the primary care providers and are more likely to be responsible for treating children and adolescents, which can be burdensome [46].

Similarly, mothers of children with other rare diseases reported greater parental stress and physical and emotional strain than fathers did [61]. Patients and caregivers with higher educational levels had higher QoL. Highly educated individuals may have better socioeconomic conditions, and parents without academic qualifications could have more difficulty achieving good metabolic control in their child [62], impairing QoL.

Patient and caregiver age were also associated with QoL: younger patients or caregivers were related to more QoL impairments. Patients of pediatric age, especially children, tend to have the greatest impairment in quality of life. This finding was observed in studies that used generic validated instruments and in those that adopted a disease-specific questionnaire (i.e., the PKU-QoL). One explanation is that younger children have more difficulty accepting and understanding PKU [50] and learning how to address their eating patterns’ specificity and the consequent differences [57]. Additionally, until the age of 12 years, good metabolic control is required to protect the child from cognitive damage. Hence, the threshold Phe concentration in young children is lower than that in other age groups [4], requiring stricter diet adherence. Moreover, in a study with patients aged between 8 and 23 years, the feeling of being different and the fear of stigmatization on occasions when food is shared were particularly emphasized by adolescents [56].

The parents of younger patients also reported the worst QoL. Younger children need more parental care and support than older children do. In the case of phenylketonuria, the younger the patient is, the greater the need for continuous supervision and management of the diet [38, 41]. Additionally, as time passes, parents gain more experience managing the disease, which may be associated with improved QoL among older parents [37].

The findings of this scoping review suggest that variables associated with the disease play crucial roles in a patient’s QoL. Treatment adherence (measured by blood Phe levels), time on treatment, and tolerance to phenylalanine were significant factors. These findings indicate that, in addition to protection from neurocognitive damage, treatment adherence can be a protective factor against impairments in quality of life. Similar findings were reported among patients with other metabolic diseases (e.g., diabetes) [63]. For PKU, early-treated diet-adherent subjects had fewer medical problems, such as eczema, asthma, and headache, than did patients who discontinued their diet early [64], which can be detrimental to their quality of life. Many negative correlations between elevated phenylalanine levels and quality of life domains reinforce the importance of treatment maintenance. Aitkenhead et al. [8] reported that partially adherent patients had poorer QoL than did those in the fully compliant and fully non-compliant groups and explained that partially adherent patients seem to have more struggles with dietary management. Adults and parents of children had difficulty with dietary management, which required significant effort, and many parents even changed their way of working or stopped working to care for patients [59]. To summarize, dietary treatment is a factor that significantly influences patients’ quality of life, with those diagnosed with classic PKU tending to have a more significant burden because the severity of the disease determines the allowed daily amount of Phe intake.

According to the review, psychosocial factors significantly influenced the quality of life of caregivers of phenylketonuria patients. Mental health problems, especially stress, have been described in several studies as a source of impairment of QoL. Given that high incidences of stress, anxiety, and depression are frequently reported in this population [43, 45], health care teams must be open to adopting a family-centered approach, including parents’/caregivers’ specific demands. Offering psychological support that includes stress management related to the disease may be beneficial, considering that emotional support mediates the association between perceived stress and parental QoL [38].

Our search revealed a significant gap in interventions specifically designed to enhance the quality of life of PKU patients. Existing interventions in this population have focused primarily on knowledge enhancement about treatment [65, 66], as it is known to correlate with improved metabolic control [62]. A comprehensive approach that combines knowledge enhancement and skills training in disease management and social skills could hypothetically yield significant benefits. These aspects of a patient’s life (e.g., disease management [treatment adherence, attitudes toward diet], and social skills [social support, emotional support]) have been identified in the literature as key factors influencing quality of life. Their absence can potentially damage QOL. As a suggestion, future interventions should address training competencies related to disease management and social skills to prevent damage in the relational aspects of life.

### Strengths and limitations

The present work is the first literature review, including studies of patients of all ages and caregivers, and offers an updated overview of the quality of life in patients with PKU disease. The extensive search performed in several databases is one of the strengths of this scoping review. Additionally, all of the search processes, article selection, and data extraction were conducted following the tools’ criteria with scientific rigor.

Although this work primarily addresses the scientific community involved in PKU, it can still offer valuable insights for patients and caregivers. Its findings validate the challenges the PKU community faces, acknowledging the significant impact the disease has on quality of life, especially with respect to social and emotional well-being. This review emphasizes the critical role of social and emotional support in improving caregivers’ quality of life, encouraging them to seek support networks. Furthermore, this review highlights the necessity of a comprehensive approach to PKU management that encompasses mental health and psychosocial care in addition to diet. Although the review is technical, its findings underscore the importance of emotional and social well-being in PKU management, which resonates with the community.

On the other hand, a few review limitations should be disclosed. Although self-report measures are crucial in assessing quality of life, as they allow individuals to express their perspectives and experiences, providing valuable insights into how the disease impacts life, the plurality of measures considered to evaluate QoL in the different studies may limit and make comparisons of QoL across individuals, populations, and studies difficult. Future studies should prioritize choosing well-validated and international measures to obtain more precise and comparable information on the impacts of PKU on QoL.

Finally, only quantitative studies published in English or Portuguese were included in the analysis, so relevant reports in other languages may not have been considered.

## Conclusions

Quality of life is a complex construct, and it is an important outcome for patients with rare diseases such as PKU. The QoL of patients with PKU varies on the basis of patient characteristics (i.e., sex, age, education level), disease severity and type of PKU, cognitive functioning and mental health, adherence to treatment, and metabolic control (i.e., better metabolic control, higher QoL). The findings of this review indicate that pediatric PKU patients are more vulnerable to QoL impairment than adult patients are (e.g., the impact is generally more pronounced in specific domains such as fatigue, emotional impact, and dietary management). Among all the patients, disease-related factors (i.e., severe dietary restrictions or poor metabolic control) had the most frequently reported associations with overall QoL and its dimensions (e.g., emotional and physical health).

On the other hand, caregivers, particularly mothers, report lower QoL than normative data do, which is often influenced by factors such as stress, parenting challenges, income level, and social support. Caregivers of younger children with PKU tend to report lower QoL than do those of older children. Factors such as parental education, social support, and emotional coping significantly affect caregivers’ QoL, with higher levels of education and support correlating with better QoL outcomes.

The QoL of adult patients assessed by generic measures did not differ from normative data or the control group. However, differences were evident within subgroups on the basis of treatment adherence; partially adherent patients often reported lower QoL. Furthermore, measurements with disease-specific questionnaires revealed negative impacts on QoL associated with treatment in all age groups of patients and caregivers. Therefore, future studies with the PKU population should consider combining short generic instruments and disease-specific instruments, such as the PKU-QoL, to evaluate QoL. Thus, as most of the variables related to QoL are modifiable factors (e.g., disease knowledge, cognitive skills, adherence behavior, social skills, coping with stigma, disease management, attitudes toward diet and nutritional knowledge) and can be manipulated in interventions, the PKU population may benefit from future interventions focused on those factors and psychosocial support.

Overall, the studies highlight the complexity of managing PKU and its impact on the QoL of both patients and their caregivers, emphasizing the importance of metabolic control, adherence to treatment, and supportive interventions to enhance QoL.

## Electronic supplementary material

Below is the link to the electronic supplementary material.

Supplementary Material 1

## Data Availability

All datasets supporting the results reported in this article are arranged on the text and supplement material.

## Abbreviations

BH4: Tetrahydrobiopterin
BVS: Virtual Health Library
CINAHL: Cumulative Index to Nursing and Allied Health Literature
IEM: Inborn error of metabolism
Phe: Phenylalanine
PKU: Phenylketonuria
PRISMA-ScR: Preferred Reporting Items Checklist for Systematic Reviews and Meta-Analyses Extension for Scoping Reviews
QoL: Quality of life
SF-36: Medical Outcomes Short-Form Health Survey 36 items
WHO: World Health Organization
WHOQOL: The World Health Organization Quality of Life Questionnaire

## Footnotes

1 Two publications included the same participants [39, 54], so only the publication with the complete sample results on QoL was included [39].
