# Supplementary material for: What is known about patients’ quality of life with Phenylketonuria and their caregivers? A scoping review
Source: Orphanet J Rare Dis. 2024 Oct 28;19:402. doi: 10.1186/s13023-024-03422-4 (PMC11520463; doi:10.1186/s13023-024-03422-4)
Supplement: Supplementary file 1 — Supplementary Material 1 [file 13023_2024_3422_MOESM1_ESM.docx]

**Additional file**

Three supplementary summary tables are presented here as additional files detailing the information on the quality of life of patients and caregivers with PKU. Table S1 summarises the studies on the quality of life of adult patients with PKU. Table S2 summarises the studies on the quality of life of pediatric patients with PKU. Finally, Table S3 summarises the studies on the quality of life of caregivers of patients with PKU.

**Table S1** Studies with adult samples

| Authors, year [Reference number] | Instrument | Were there significant differences in total QoL or its domains? | If yes, in which domain? | |
| --- | --- | --- | --- | --- |
|  |  |  | **Significantly lower scores/impairment in QoL** | **Significantly higher scores** |
| Aitkenhead et al., 2021 [8] | SF-36 | No | - | - |
| Barta et al., 2020 [35] | PKU-QoL | N/A¹ | - | - |
| Bik-Multanowski et al., 2008 [52] | Psychological General Well-Being Index | N/A² | - | - |
| Bosch et al., 2007 [48] | RAND-36 Health Survey  Cognitive Scale of the TNO-AZL Adult Quality of Life (TAAQoL) questionnaire | No | - | - |
| Klimek et al., 2020 [34] | Ad hoc questionnaire | N/A³ | - | - |
| Palermo et al., 2020 [31] | SF-36 | No | - | - |
| Bosch et al., 2015 [28] | SF-36  PKU-QoL | Yes | Mental domains (vitality, social functioning, role-emotional limitations, mental health) | Physical domains (physical functioning, role-physical limitations, bodily pain, general health) |
| Cazzorla et al., 2014 [40] | WHOQOL-100 | No | - | - |
| Cotugno et al., 2011 [27] | SF-36 | No | - | - |
| Das et al., 2014 [29] | Alltagsleben questionnaire | No | - | - |
| Demirdas et al., 2013 [33] | TNO-AZL Adult Quality of Life (TAAQoL) questionnaire | Yes | Cognitive functioning | - |
| Hujibregts et al., 2018 [49] | TNO-AZL Adult Quality of Life (TAAQoL) questionnaire | Yes | Cognitive functioning, depressive emotions, and aggressiveness domains | - |
| Gassió et al., 2003 [30] | Ad hoc questionnaire | N/A² | - | - |
| Simon et al., 2008 [32] | Profile of Quality of Life in the Chronically Ill Questionnaire | No | - | - |

Notes:

N/A: Not Applicable

¹ It used a specific questionnaire without the possibility of comparison with the general population and/or normative data (PKU Quality of Life)

² Authors did not compare results with data from the general population but accessed changes on QoL in intragroup (pre and post-)

³ It was used as an ad hoc questionnaire without the possibility of comparison with the general population and/or normative data

**Table S2** Studies with pediatric samples

| Authors, year [Reference number] | Instrument | Were there significant differences in total QoL or its domains? | If yes, in which domain? | |
| --- | --- | --- | --- | --- |
|  |  |  | **Significantly lower scores/impairment in QoL** | **Significantly higher scores** |
| Alptekin et al., 2018 [50] | PKU-QoL | N/A¹ | - | - |
| Bosch et al., 2015 [28] | Pediatric Quality-of-Life Inventory  PKU-QoL | No | - | - |
| Cazzorla et al., 2014 [40] | Pediatric Quality of Life Inventory | No | - | - |
| Cotugno et al., 2011 [27] | Child Health Questionnaire (Child and Parent Form) | Yes | Global health, physical functioning, role social limitation-emotional, role social limitation-physical, behavior, mental health, parental impact-emotional, parental impact-time, family activities | - |
| Demirdas et al., 2013 [33] | Pediatric Quality of Life Inventory  DISABKIDS chronic generic module | Yes | - | Total scale and psychosocial functioning (in adolescents), physical functioning (in children) |
| Hujibregts et al., 2018 [49] | TNO-AZL Questionnaire for Children's Health  Related Quality of Life (Child and Parent Form) | Yes | Autonomy dimension (in children, rated by parents) cognitive functioning (in adolescents, self-report) | - |
| Das et al., 2014 [29] | Alltagsleben (AL) questionnaire | No | - | - |
| Gassió et al., 2003 [30] | Ad hoc questionnaire | N/A² | - | - |
| Simon et al., 2008 [32] | Profile of Quality of Life in the Chronically Ill Questionnaire | No | - | - |
| Landolt et al., 2002 [44] | TNO-AZL Child Quality of Life Questionnaire (Parent Form) | Yes | Positive emotions domain (on the proxy report) | - |
| Thimm et al., 2013 [36] | KINDL-R questionnaire | Yes | Everyday functioning (school or nursery school, on the proxy report) | - |
| Vieira Neto et al., 2017 [42] | Pediatric Quality of Life Inventory | Yes | Physical health, emotional functioning, social functioning, school functioning, and psychosocial health | - |

Notes:

N/A: Not Applicable

¹ It used a specific questionnaire without the possibility of comparison with the general population and/or normative data (PKU Quality of Life)

² Authors did not compare results with data from the general population but accessed changes on QoL in intragroup (pre and post-)

**Table S3** Studies with caregivers in the sample

| Authors, year [reference number] | Instrument | Were there significant differences in total QoL or its domains? | If yes, in which domain? | |
| --- | --- | --- | --- | --- |
|  |  |  | **Significantly lower scores/impairment in QoL** | **Significantly higher scores** |
| Ben Abdelaziz et al, 2020 [43] | SF-36 | Yes | - | Physical component subscore |
| Bosch et al., 2015 [28] | Child Health Questionnaire-Parent Form 28  PKU-QoL | Yes | Emotional parental impact domain | - |
| Etemad et al., 2020 [37] | WHOQOL-BREF | Yes | Psychological health, environment, and social relationships domains | - |
| Fidika et al., 2013 [41] | The Ulm Quality of life Inventory for Parents of chronically ill children | N/A¹ | - | - |
| Iakovou & Schulpis, 2020 [53] | Ad hoc questionnaire | N/A² | - | - |
| Iakovou et al., 2019 [47] | Ad hoc questionnaire | N/A³ | - | - |
| Irannejad et al., 2018 [46] | SF-36 | Yes | Total score^5^ | - |
| Mahmoudi-Gharaei et al., 2011 [45] | WHOQOL-BREF | Yes | Psychological health, physical health, social relationships, and environment domains | - |
| Mitchell et al., 2021 [54] | PKU-QoL | N/A^4^ | - | - |
| Morawska et al., 2020 [39] | PKU-QoL | N/A^4^ | - | - |
| ten Hoedt et al., 2011 [38] | TNO-AZL Questionnaire for Adult's Health-related Quality of Life (TAAQoL) | No | - | - |

Notes:

N/A: Not Applicable

¹ It used a questionnaire without normative data (The Ulm Quality of Life Inventory for Parents of chronically ill children)

² Interventional study

³ It was used as an ad hoc questionnaire without the possibility of comparison with the general population and/or normative data

^4^ It used a specific questionnaire, without the possibility of comparison with the general population and/or normative data (PKU Quality of Life)

^5^ Authors do not analyze by domain but report the mean score of QoL as lower than the average level.
